# Same risk – different profile? Identification of different risk profiles for dementia in the German National Cohort NAKO

**DOI:** 10.1002/alz70860_106369

**Published:** 2025-12-23

**Authors:** Felix Georg Wittmann, Susanne Röhr, Sebastian Köhler, Niels Janssen, Melanie Luppa, Michael Wagner, Luca Kleineidam, Klaus Berger, Alexander Pabst, Steffi G. Riedel‐Heller

**Affiliations:** ^1^ Institute of Social Medicine, Occupational Health and Public Health (ISAP), Medical Faculty, University of Leipzig, Leipzig, Germany; ^2^ Global Brain Health Institute (GBHI), Trinity College Dublin, Dublin, Ireland; ^3^ School of Psychology, Massey University, Auckland, Auckland, New Zealand; ^4^ Alzheimer Center Limburg, Mental Health and Neuroscience Research Institute, Maastricht University, Maastricht, Netherlands; ^5^ Department of Psychiatry and Neuropsychology, Alzheimer Centrum Limburg, Mental Health and Neuroscience Research Institute (MHeNs), Maastricht University, Maastricht, Netherlands; ^6^ German Center for Neurodegenerative Diseases (DZNE), Bonn, Germany; ^7^ Department of Neurodegenerative Diseases and Geriatric Psychiatry, University of Bonn Medical Center, Bonn, Germany; ^8^ Institute of Epidemiology and Social Medicine, University of Muenster, Muenster, Germany

## Abstract

**Background:**

Risk and protective factors for dementia are well established. Multidomain lifestyle interventions have shown promise in reducing dementia risk, yet their effectiveness often varies across predictors and subgroups. To enhance prevention strategies, it is crucial to tailor interventions more effectively. While research is focusing on single risk factors or sum scores, evidence on more specific risk profiles is lacking. The LIfestyle for BRAin Health (LIBRA) index is a standardized index to calculate dementia risk by integrating modifiable risk and protective factors. We aimed to identify distinct risk profiles for dementia based on the LIBRA factors.

**Method:**

Using a three‐step procedure, a Latent Class Analysis was conducted with *n* = 106,192 participants of the German National Cohort (NAKO; aged 40–75, mean age 51.4 years, 49.4% women) to identify distinct classes (i.e. risk profiles). Ten LIBRA factors (coronary heart disease, hypertension, diabetes, hypercholesterolemia, depression, obesity, smoking, alcohol consumption, physical inactivity, and low social participation) were used as indicators, followed by analyses of sociodemographic predictors of class membership and class‐specific differences in cognitive functioning accounting for classification uncertainty.

**Result:**

A latent four‐class model fitted the data best: The largest class (>60%) represents a low‐risk group with low probabilities across all factors. A second class (∼16%) was defined by cardiometabolic risks (high probabilities of hypercholesterolemia, hypertension and comparatively high values for heart disease and diabetes). A third class (14%) is mainly defined by low social participation but also high smoking rates and comparatively higher physical inactivity, alcohol intake, and depression. The fourth and smallest class (∼8%) consisted entirely of individuals with obesity and high hypertension probability. Results are preliminary and will be detailed regarding predictors and cognitive functioning at the conference.

**Conclusion:**

Identifying four distinct dementia risk profiles offers the potential for more targeted prevention strategies. Instead of a one‐size‐fits‐all approach, tailored interventions may yield greater benefits for individuals characterized by a specific high‐risk profile. Highlighting the importance of replication and validation in future studies, these findings have the potential to reshape intervention study designs and public health campaigns. Early interventions could be better tailored, ultimately contributing to more effective dementia risk reduction.

